# Assessing the use of urinary creatinine excretion rate to estimate urine output in lactating cows

**DOI:** 10.3168/jdsc.2024-0736

**Published:** 2025-05-12

**Authors:** K. Park, J. Kim, H. Hu, C. Lee

**Affiliations:** Department of Animal Sciences, The Ohio State University, Wooster, OH 44691

## Abstract

Creatinine concentration in urine is often used to estimate urine output in cows. Studies have commonly used a fixed coefficient for the creatinine excretion rate, although this coefficient varies among studies. However, 29 mg/kg BW·d^−1^ is the most popular value. The current study determined the coefficient for the creatinine excretion rate of individual cows and validated the use of a fixed coefficient to estimate urine output. Two experiments were conducted with 8 cows in each experiment. The experiments were conducted in a replicated 4 × 4 Latin square design. In experiment 1, dietary treatments were 2 levels of RDP with or without isoacids supplementation in 2 × 2 factorial arrangement. In experiment 2, diets at 2 levels of DCAD supplemented with saturated fatty acids or a Met analog were fed to cows as dietary treatments. Total urine was collected over multiple days in each period, followed by BW measurements before feeding on 2 consecutive days. Daily subsamples of urine were composited by cow and period and assayed for creatinine concentration. The coefficient for the creatinine excretion rate was calculated by daily urine creatinine excretion (mg/d) divided by BW for individual cows. Data from each experiment were analyzed using the MIXED procedure of SAS, where fixed effects of period, diet, and their interaction and random effects of cow within square were included. Additionally, data from both experiments were combined and analyzed using the MIXED procedure of SAS, where cow within trial and period within trial were random effects and trial was the fixed effect. The experimental period and dietary treatments did not affect total urinary creatinine excretion or the coefficient within each experiment, although the periods influenced milk yield and BW. There was no difference in DMI and milk yield between the experiments, but BW differed (672 vs. 746 kg). In addition, urinary creatinine excretion (12.8 vs. 17.8 g/d) and the coefficient (19.1 vs. 23.9 mg/kg BW·d^−1^) were greater in experiment 2 compared with experiment 1. A linear regression analysis between BW and urinary creatinine excretion was conducted using the MIXED procedure of SAS (experiment as a random effect). The coefficient of 22.0 mg/kg BW·d^−1^ was obtained from the analysis and this was lower than the 29 mg/kg BW·d^−1^ value that has been commonly used in the literature. In conclusion, the coefficient varied among cows, likely because BW does not represent muscle mass. Therefore, caution is needed when urine output is estimated using a fixed coefficient in an experiment.

## Highlights

• The urinary creatinine excretion rate varies highly among cows.
• The urinary creatinine excretion rate obtained was 22.0 mg/kg of BW per day.
• Accounting for muscle mass may reduce the variation in the creatinine excretion coefficient.

## Graphical Abstract

**Figure:**
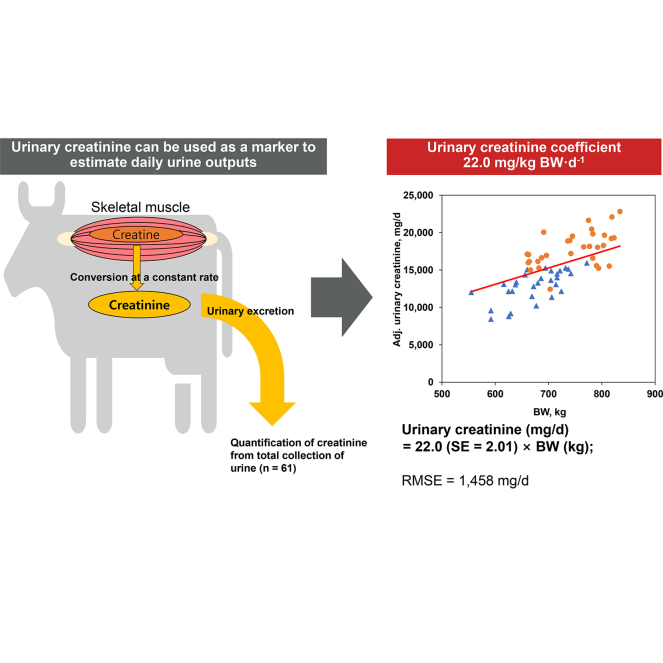
**Summary:** Urinary creatinine concentration has been used to estimate urine output in lactating cows where fixed coefficients for creatinine excretion have been used and vary among studies. The objective of this study was to determine the coefficient for the creatinine excretion rate of individual cows and evaluate its variability. Two total-collection experiments were conducted with 8 cows in each experiment. We determined variability for the coefficient among cows and between studies. A linear regression analysis was conducted with the data from the 2 studies and obtained the coefficient of 22.0 mg/kg body weight (BW)·day^-1^. In conclusion, the coefficient varied among cows, likely because BW does not accurately reflect muscle mass. Therefore, caution is needed when urine output is estimated using a fixed coefficient in an experiment. SE = standard error; RMSE = root mean squared error.

The quantification of daily urine output for individual cows is often needed in nutrition research to evaluate diet formulations on nutrient utilization and excretion. Total collection of urine is the gold standard to accurately determine daily urine output of cows. However, total collection of urine for individual cows is laborious and often applicable to an experiment that uses a small number of animals. As an alternative indirect method, urinary creatinine concentration has been used as a marker to estimate daily urine output (Valadares et al., 1999; Tebbe and Weiss, 2018; Lee et al., 2019). The principal behind the urinary creatinine concentration as a marker is based on the assumption that creatinine is formed at a constant rate through spontaneous and irreversible conversion of creatine in skeletal muscles (Bloch and Schoenheimer, 1939; Wyss and Kaddurah-Daouk, 2000) and is not significantly influenced by diets (Susmel et al., 1995; Moorby et al., 2006; Chizzotti et al., 2008). If creatinine is formed and excreted in urine at a constant rate, the amount of creatinine formed and excreted in urine should be correlated with the muscle mass. Then, if the body muscle mass and creatinine excretion rate per unit of muscle mass are known, the volume of urine can be estimated using urinary creatinine concentration. Currently, BW has been used as a proxy for the muscle mass. Although 29 mg/kg of BW·d^−1^ of the creatinine excretion rate as the coefficient has been often used (Valadares et al., 1999), various coefficients have been observed (22.8, 25.5, and 27.3 mg/kg BW·d^−1^) in previous studies (Lee et al., 2019; Letelier et al., 2024; Asai et al., 2005). In addition, unrealistic N retention (>10% of N intake) was often observed in mid-lactation cows in our previous studies (C. Lee, The Ohio State University, Wooster, OH, unpublished) likely due to underestimation of urinary N excretion (15% of N intake) when 29 mg/kg of BW·d^−1^ of the coefficient was used. This led us to a hypothesis that the coefficient for the urinary creatinine excretion rate is not fixed and varies depending on various factors. This study aimed to determine animals and time periods as potential factors that influence the coefficient for the urinary creatinine excretion rate and confirm that dietary intervention is not a factor.

Two experiments were conducted to determine the coefficient. Experiments 1 and 2 were conducted at the Krauss Dairy Research Center (The Ohio State University, Wooster, OH), and all procedures and animal care were approved by the Institutional Animal Care and Use Committee at The Ohio State University (2022A00000065 and 2024A00000029, respectively). Experiment 1 used 8 Holstein cows (mean ± SD; 214 ± 41 DIM) in a replicated balanced 4 × 4 Latin square design. The following dietary treatments were applied: a low RDP diet (9% of diet DM), the low RDP diet supplemented with iso-butyrate and 2-methyl butyrate (0.216% of diet DM), a high RDP diet (11% of diet DM), and the high RDP diet supplemented with iso-butyrate and 2-methyl butyrate. The composition of the diets was 50% corn silage, 6% alfalfa silage, and 44% concentrate on a DM basis (15% or 17% CP, 29% NDF, and 30% starch on a DM basis). The experiment consisted of 4 experimental periods with 28 d in each period. Total urine was collected for the last 3 d in each period using a urinary catheter (silicone-elastomer coated latex Foley catheters, Medline Industries Inc.). The catheter was inserted into the individual cows a day before starting the collection, and urine via the catheter was collected into a container containing 2.0 L of 2*M* H_2_SO_4_. Total collection of urine was conducted daily over 3 d. In each day, a subsample of daily total urine was collected and frozen at −20°C until analyses. Experiment 2 used 8 Holstein cows (mean ± SD; 202 ± 16 DIM) in a replicated balanced 4 × 4 Latin square design. Four dietary treatments were used as follows: diet with a fat supplement enriched with C16:0 and C18:0 fatty acids (200 mEq/kg DM of DCAD), a diet with reduced DCAD (5 mEq/kg DM), the reduced DCAD diet with a fat supplement enriched with C16:0, and the reduced DCAD diet supplemented with 2-hydroxy 4-methylthio butanoic acid. The composition of the diets was 29% corn silage, 24% alfalfa silage, and 47% concentrate (16% CP, 32% NDF, and 25% starch) on a DM basis. The experiment consisted of 4 experimental periods with 21 d in each period. Total urine was collected for the last 4 d in each period as described in experiment 1. Daily subsamples of urine were composited in proportion to daily excretion by cow and period within the experiment, and the composite samples were assayed for creatinine concentration using a colorimetric reaction method (500701, Cayman Chemical; Heinegård and Tiderström, 1973). All the samples were analyzed in duplicate, and only results with a CV below 5% were accepted. If the CV exceeded 5%, the samples were reanalyzed until it fell below the threshold. The coefficient for the creatinine excretion rate of individual cows was calculated by the following equation:

$$\begin{array}{l} coefficient\;\left(mg/kg\;BW\cdot d^{-1}\right)\\ =\frac{urine\;volume\;\left(kg/d\right)\times urinary\;creatinine\;concentration\;\left(mg/kg\right)}{BW\;\left(kg\right)}. \end{array}$$

During the experiments, milk yield and DMI during the last week in each period were recorded, and BW was measured immediately before feeding for 2 continuous days after the last day of total urine collection. Statistical analysis was conducted using the MIXED procedure of SAS (version 9.4; SAS Institute Inc.). All data from 2 cows in period 4 of experiment 1 were excluded because one cow had an abomasal displacement and another had an unknown health issue (depressed feed intake and milk yield). Urine data (urine output and creatinine concentration, excretion, and coefficient) of 1 cow in period 3 of experiment 2 were excluded due to urine contamination during collection. Therefore, a total of 30 and 32 observations were obtained for DMI, production, and BW from experiments 1 and 2, respectively, and 30 and 31 observations for urine output, creatinine concentration and excretion, and coefficient from experiments 1 and 2, respectively, for statistical analysis. Data were analyzed within each experiment where fixed effects of period, diet, and their interaction and random effects of square and cow within square were included. Then, the data from experiments 1 and 2 were combined and analyzed to assess the differences in those variables between the two experiments using the MIXED model with a fixed effect of trial and random effects of cow within square × trial and period within trial. The data of BW and creatinine excretion were also analyzed for a linear regression using the MIXED procedure, where random effects of trial, cow within trial, and period within trial were included in the regression model. For the graphical presentation, y values (urinary creatinine excretion) were adjusted due to random effects using the predicted and residual values (St-Pierre, 2001). Significance was declared when *P* < 0.05, and tendency was considered when 0.05 ≤ *P* < 0.10.

We used a total of 16 cows in the experiments and obtained 61 observations to assess the coefficient for the creatinine excretion rate. The characteristics and performance of cows used in the experiments varied: (mean ± SD) lactation number from 2 to 4 (2.4 ± 0.70), DIM from 172 to 348 d (245 ± 42), milk yield from 22.1 to 50.0 kg/d (38.8 ± 5.2), DMI from 16.8 to 35.6 kg/d (27.1 ± 3.4), and BW from 555 to 834 kg (711 ± 66). Accordingly, the daily output of urine (14.0 to 51.2 kg/d; 32.7 ± 7.0) varied as well.

In experiment 1, no effect of dietary treatments was observed on DMI, milk yield, BW, urine output, and urinary creatinine excretion and coefficient (Table 1). The period effect was observed (*P* < 0.01) only for milk yield and BW, and no interactions between dietary treatments and periods were observed. Generally, the period effect reflected a trend of decreasing milk yield and increasing BW from period 1 to 4. In experiment 2, DMI was affected (*P* = 0.04) by dietary treatments, and milk yield tended to be affected (*P* = 0.08) by dietary treatments. Milk yield, DMI, and BW were affected (*P* < 0.01) by periods where a trend of decreasing DMI and milk yield and increasing BW from period 1 to 4 was observed. However, urinary creatinine excretion and coefficient were not affected by periods. No interaction between periods and dietary treatments was observed in experiments 1 and 2.

**Table 1:** Dry matter intake, milk yield, BW, urine excretion, urinary creatinine excretion, and creatinine coefficient observed from lactating cows in 2 replicated 4 × 4 Latin square design experiments^1^

Item	Period				SEM	*P-*value^2^		
	1	2	3	4		Per	Diet	Int
Experiment 1								
DMI, kg/d	27.3	27.3	25.4	25.9	1.25	0.20	0.78	0.62
Milk yield, kg/d	42.4^a^	40.9^ab^	34.4^c^	36.9^bc^	2.22	<0.01	0.65	0.98
BW, kg	650^b^	674^a^	677^a^	691^a^	20.6	<0.01	0.66	0.64
Urine, kg/d	30.1	32.9	29.0	31.5	1.50	0.33	0.54	0.13
Urinary creatinine, g/d	13.1	12.4	12.5	13.6	0.82	0.51	0.41	0.53
Coefficient^3^	20.2	18.3	18.4	19.7	0.99	0.41	0.58	0.53
Experiment 2								
DMI, kg/d	30.1^a^	26.5^c^	27.8^b^	26.6^c^	1.31	<0.01	0.04	0.77
Milk yield, kg/d	40.1^a^	38.6^b^	39.8^ab^	35.4^c^	1.55	<0.01	0.08	0.12
BW, kg	738^b^	743^b^	741^ab^	761^a^	23.7	<0.01	0.88	0.81
Urine, kg/d	33.4	34.1	34.5	37.3	2.34	0.29	0.12	0.70
Urinary creatinine, g/d	17.4	17.2	19.2	17.7	0.69	0.17	0.69	0.18
Coefficient^3^	23.6	23.2	25.6	23.2	0.79	0.19	0.57	0.17

a–c Within a row, values with different superscripts differ significantly.

1 Four dietary treatments were applied to cows in each experiment and the diets between experiments 1 and 2 were different.

2 Per = the effect of period; Diet = the effect of diets; Int = interaction between periods and diets.

3 Daily creatinine excretion rate per kilogram of BW (mg/kg BW·d^−1^).

When production and urine parameters were compared between the experiments (Table 2), milk yield and DMI were not different. Body weights of cows were greater (*P* = 0.04) for cows in experiment 2 compared with those in experiment 1. Daily urine output was not different, but urinary creatinine excretion and the coefficient were greater (*P* < 0.01) for cows in experiment 2 compared with those in experiment 1. To obtain the coefficient from the combined data from experiments 1 and 2, a linear regression between BW and urinary creatinine excretion was conducted and is shown in Figure 1. The intercept of the regression was not different from 0 and was removed (Tebbe and Weiss, 2018) from the equation (intercept = −1,363, SE = 3,884, *P* = 0.79). The root mean squared error (**RMSE**) was 1,458 mg/d (9.5% of the mean). As a result, the coefficient of 22.0 (mg/kg BW·d^−1^) was obtained from 61 observations.

**Table 2:** Experiment effects on DMI, milk yield, BW, urine excretion, urinary creatinine excretion, and coefficient for the urinary creatinine rate

Item	Experiment		SEM	*P*-value
	1	2		
DMI, kg/d	26.4	27.7	1.23	0.47
Milk yield, kg/d	38.6	38.5	2.13	0.98
BW, kg	672	746	20.6	0.04
Urine, kg/d	31.0	34.8	1.79	0.19
Urinary creatinine, g/d	12.8	17.8	0.64	<0.01
Coefficient^1^	19.1	23.9	0.62	<0.01

1 Daily creatinine excretion rate per kg of BW (mg/kg BW·d^−1^).

**Figure 1:**
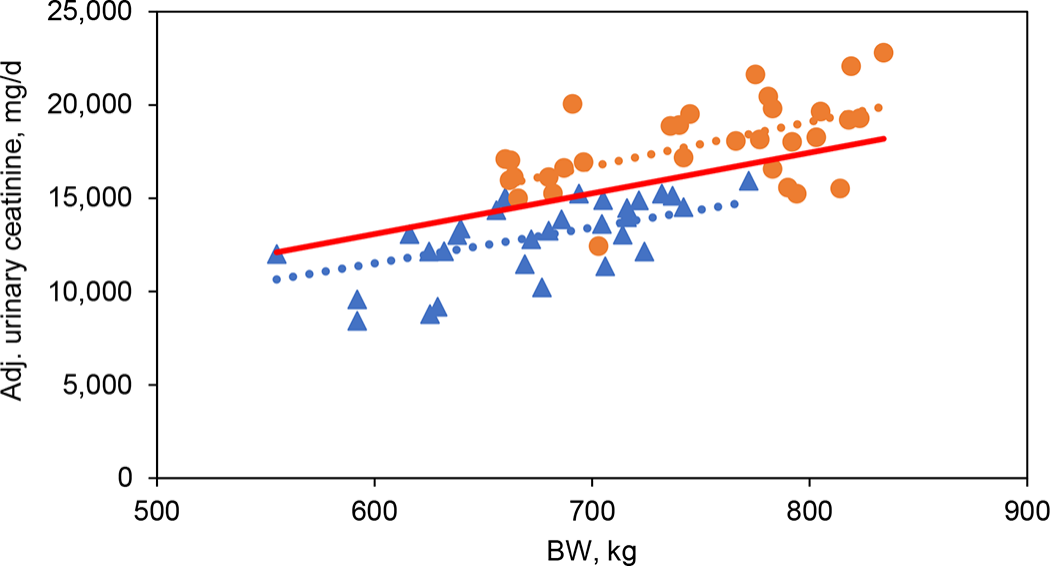
A linear regression between BW (kg) and urinary creatinine excretion (mg/d adjusted from random effects; red line). The triangle and circle symbols represent observations in experiments 1 (dotted blue line) and 2 (dotted orange line), respectively. Urinary creatinine (mg/d) = 22.0 (SE = 2.01) × BW (kg); RMSE = 1,458 mg/d (9.5% of the mean). The intercept of the regression was not different from 0 and was removed in the equation (intercept = −1,363, SE = 3,884, *P* = 0.79).

The period effects on milk yield and BW in experiment 1 and DMI, milk yield, and BW in experiment 2 were expected because they change as the lactation progresses (Pollott, 2000). No effect of dietary treatments on urinary creatinine excretion and coefficient in experiments 1 and 2 suggests that the diets used in those experiments did not alter creatinine metabolism within the experiment. The absence of the diet effects on creatinine excretion and coefficient is in line with previous studies (Susmel et al., 1995; Moorby et al., 2006; Chizzotti et al., 2008). We did not find any period effect on urine output, creatinine excretion, and coefficient in experiments 1 and 2. This suggests that creatinine metabolism was not affected by time during the experiments. Interestingly, urinary creatinine excretion did not change during the experiment although BW increased as the experiment progressed, that is, there was a period effect. Because the amount of creatinine formation and excretion depends on the mass of body muscles (Wyss and Kaddurah-Daouk, 2000), our results suggest that the BW changes of the mid- and late-lactation cows in the current experiments were likely attributed mainly to changes in fat mass rather than muscle mass. Indeed, Andrew et al. (1994) observed that body fat changed significantly from prepartum to late lactation (89.9, 47.5, and 80.9 kg of total fat at prepartum, early lactation, and late lactation, respectively) and total protein did not change (77.8, 81.1, and 85.9 kg), indirectly supporting small changes of the size of body muscles during the entire lactation stages.

Although urine output did not differ, we observed clear differences in urinary creatinine excretion between experiments 1 and 2. The difference, at least in part, was attributed to different BW between the experiments. However, BW did not fully explain the difference because the coefficient was also greater for cows in experiment 2 compared with that in experiment 1. Then, the questions that arise according to the results are as follows: (1) why the average coefficients were different (19.1 vs. 23.9 mg/kg BW·d^−1^) between experiments 1 and 2, and (2) why the average coefficient obtained from the current 2 experiments was lower (22 vs. 29 mg/kg BW·d^−1^) than the coefficient that has been often used in the literature. In the literature, a fixed coefficient has been used within a study and the coefficient used varies from 22.8 to 29 mg/kg BW·d^−1^ (Valadares et al., 1999; Asai et al., 2005). Although a fixed coefficient was used within a study, Tebbe and Weiss (2018) and Lee et al. (2019) pointed out the large variation of the coefficient between cows (20 to 36 and 16.7 to 34.5, respectively). Likewise, a large variation of the coefficient was observed in the current experiments (14 to 29 mg/kg BW·d^−1^). The large cow-to-cow variation indicates that the use of a fixed coefficient to estimate urine output for individual cows in an experiment could result in the estimation of inaccurate urine output and nutrient excretions. We concluded that the cow-to-cow variation is the major factor that led to the difference in the coefficient between experiments 1 and 2 and the coefficient being lower in the current experiments compared with that often used in the literature. As described earlier, the large cow-to-cow variation of the coefficient is likely explained mainly by BW that does not fully represent the mass of skeletal muscles. Indeed, BW was negatively correlated with urinary creatinine excretion per kilogram of BW (Chizzotti et al., 2008), suggesting that the proportion of muscle was diluted by fat to an extent as BW increased. If the proportion of muscle mass in BW varied among cows and this was the major factor causing the large cow-to-cow variation of the coefficient, obtaining the coefficient based on the unit of muscle mass instead of BW should be able to resolve the large variation problem. However, because the accurate measurement of the skeletal muscle mass for individual cows is not possible, studies are needed to develop a way for correction of BW to reflect more toward body muscle, that is, correction for the degree of fatness or adjustment based on any measures that could indirectly indicate the body muscle such as BCS, plasma creatinine, or ultrasonographic measurement of muscle thickness (Bruckmaier et al., 1998; Megahed et al., 2019).

The lactation stage (i.e., DIM) cannot be ruled out as a factor causing the large variation of the coefficient among cows. However, the stage of lactation is likely a minor factor at least for mid- and late-lactation cows because we observed no period effect on the coefficient within the experiment. In addition, cows were at similar DIM between experiments 1 (mean ± SD; 213 ± 41d) and 2 (202 ± 15d) but had different coefficients. Furthermore, in our previous study (Lee et al., 2019), DIM of cows was 205 ± 19 d and the average coefficient was 27.3 mg/kg BW·d^−1^. The number of lactations might be another factor. All cows in experiment 1 were second lactation and 2.75 ± 0.89 (mean ± SD) in experiment 2, and our data were not sufficient to determine the lactation number as a factor causing the cow-to-cow variation of the coefficient. Additionally, other unknown experimental factors may have contributed to the large cow-to-cow variation within the experiment or to the difference in coefficients between the 2 experiments in the current study, but they could not be identified within our experimental designs.

In conclusion, the coefficient for the urinary creatinine excretion rate was not affected by dietary treatments and periods under the experimental conditions. The difference in the coefficient between the experiments is most likely explained by cow-to-cow variation. The coefficient varied from 14 to 29 mg/kg BW·d^−1^ for the cows used in the current experiments and was on average 22.0 mg/kg BW·d^−1^. Because of the large cow-to-cow variation, the use of a fixed coefficient may result in inaccurate estimation of urine output and nutrient excretion, potentially leading to wrong conclusions. Relative comparisons of urine output and nutrient excretion estimated by a fixed coefficient among treatments might be still possible in a Latin square-type experiment; however, caution is needed for results from a randomized block design. More studies are needed to obtain the coefficient calculated based on the mass of body muscle instead of BW, which should estimate urine output more accurately.
